# The League of Mercy

**Published:** 1904-10-15

**Authors:** 


### THE LEAGUE OF MERCY.

T.R.H. the Prince and Princess of Wales (Grand President and Lady Grand President) have been pleased to make the following appointments in the League of Mercy:—

H.S.H. Prince Alexander George of Teck, K.C.V.O., D.S.O., President of the Kingston-Surbiton District; His Grace the Duke of Norfolk, K.G., G.V.O., President of the Chichester District; Sir Edward D. Stern, President of the Chertsey District; the Ven. Samuel Cheetham, D.D. (Archdeacon of Rochester), and Mrs. Cheetham, President and Lady President of the Rochester District; Dr. and Mrs. A. Carson Smith, President and Lady President of the Willesden District; Mr. and Mrs. Harry Goddard, President and Lady President of the Brixton District; Mr. C. J. Lucas and Mrs. Maudslay, President and Lady President of the Horsham District; Mr. and Mrs. Thos. F. Rider, President and Lady President of the Southwark (West) District; Mr. and Mrs. George Winch, President and Lady President of the Chatham District; Mrs. Carl Meyer, Lady President of the Saffron Walden District; Mrs. Keppel, Lady President of the Worthing District.

